# Unmasking Cognitive Decline: A Case Report on the Diagnostic Challenges in Language Variant Frontotemporal Dementia

**DOI:** 10.1192/bjo.2025.10714

**Published:** 2025-06-20

**Authors:** Rishabh Chormalle

**Affiliations:** Lincolnshire Partnership Foundation Trust, Lincoln, United Kingdom

## Abstract

**Aims:** Language variant frontotemporal dementia (lvFTD) is a neurodegenerative disorder primarily affecting language, often presenting with speech and comprehension difficulties, commonly in people aged 45–65 years. LvFTD presents a perplexing diagnostic challenge, often masquerading as primary progressive aphasia (PPA) while progressively dismantling communication and cognition. Despite growing recognition of lvFTD, a critical gap remains in distinguishing its early presentation with overlapping neurodegenerative syndromes, delaying accurate diagnosis and intervention.

**Methods:** This case report highlights the challenges of diagnosing lvFTD in a patient with atypical early symptoms and the implications of late diagnosis on patient care and outcomes.

Patient in her late 50s, with background of anaemia and hypothyroidism presented with memory issues, word-finding difficulties, and trouble understanding conversations which began in early 50s, initially attributed to menopause. Symptoms worsened over time, revealing frontal lobe atrophy on brain imaging and a psychotic episode, including auditory hallucinations and paranoid delusions.

Following two self-harm attempts, detention under the Mental Health Act led to first mental health admission. Neurological investigations, including PET CT Brain, suggested a diagnosis of potential Logopenic Primary Progressive Aphasia (lvPPA) with mixed dementia and Lewy Body dementia (LBD). Neurologist review confirmed diagnosis of lvFTD. Antipsychotic trial undertaken with aripiprazole, only to stop as led to worsening behavioural symptoms. Subsequently started on mirtazapine, quetiapine, lorazepam and rivastigmine. Improvement noticed in symptomatology. Currently awaiting DAT scan for further evaluation and on waitlist of Young Onset Dementia Psychology.

**Results:** This case underscores the complex diagnostic challenges in patients with overlapping neurodegenerative and psychiatric symptoms. The patient, in her late 50s, presented with progressive language impairment, memory issues, and psychotic features including auditory hallucinations and paranoid delusions. Neuroimaging revealed frontal lobe atrophy and significant asymmetrical hypometabolism in the left frontal, temporal and parietal lobes, findings suggestive of lvPPA. However, reduced tracer activity in the occipital cortices raised the possibility of mixed dementia, potentially co-existing with LBD. These overlapping features highlight the need for a comprehensive, multidisciplinary approach to refine diagnosis and optimize management strategies.

**Conclusion:** Breaking through the diagnostic fog, this case exposes the intricate challenge of untangling overlapping neurodegenerative and psychiatric disorders. The patient’s progression from language deficits to memory loss and psychotic symptoms along with neuroimaging showing left hemispheric hypometabolism and frontal lobe atrophy, suggested lvPPA, potentially complicated by mixed dementia and probable LBD. She was diagnosed as lvFTD. This complexity calls for early multidisciplinary evaluation for prompt diagnosis and tailored intervention for improved patient outcomes.

